# Competing framings of abortion access reform campaigns in Madagascar: barriers to effective coalition building

**DOI:** 10.3389/frph.2026.1809340

**Published:** 2026-07-20

**Authors:** Mina H. Rakotoarindrasata, Eugene Andriamasy, Tamaryn L. Crankshaw, Jane Freedman

**Affiliations:** 1 SRHR Coalition of the Generation Equality Forum, Antananarivo, Madagascar; 2 Health Economics and HIV and AIDS Research Division (HEARD), University of KwaZulu-Natal, eThekwini/Durban, South Africa; 3 Jane Freedman, CRESPPA, Université Paris 8, Paris, France

**Keywords:** abortion, civil society, issue framing, legal reform, Madagascar

## Abstract

In sub-Saharan Africa, civil society organisations have a key role in putting abortion law and policy reform on the political agenda, and mobilising opinion in favour of such reform. However, the US global gag rule and the “anti-gender” movements, currently growing in strength across the world, have had a negative impact on opportunities for advocacy and coalition building across the region, restricting the space for debate, mobilisation and funding for civil society. In Madagascar, a country with one of the most restrictive abortion laws in sub-Saharan Africa, civil society mobilisations in favour of abortion law reform have been hampered by divisions within the pro-choice movement, with disagreements both on issue framing and on mobilisation tactics. Based on key informant interviews (n20) with members of civil society, journalists and politicians in Madagascar, we analyse the divisions that have emerged in pro-choice civil society and the ways that these might impact on their shared larger objective of law reform. We argue that there is a role for regional learning and exchange to help to overcome these national divisions and create stronger and more effective coalitions to advance reproductive rights.

## Introduction

Women across the world experience inequities in access to safe abortion services and this is most acutely felt in country contexts where the grounds for legal abortions are highly restricted. Restricting access to abortions does not reduce the number of abortions - it just makes them more unsafe (42, 43). And on the contrary, evidence shows that legislative reforms to liberalise Termination of Pregnancy (TOP) law, are associated with reductions in fertility and lower maternal mortality (44). In sub-Saharan Africa, pressure for law reform has been aligned with the landmark African Union's Maputo Protocol which is the first African human rights treaty to explicitly recognize abortion as a human right under certain circumstances and to which the majority of countries in sub-Saharan Africa (SSA) are signatories.

As with other gender equality issues such as gender-based violence (1), civil society organisations (CSOs) have had a key role in many countries in putting abortion law reform on the political agenda, and mobilising social movements in favour of this reform. In the African context, research has shown the ways in which civil society has been instrumental in mobilising in favour of abortion law reform in Ethiopia or Malawi for example (2–4). However, the US global gag rule and the “anti-gender” movements which are currently growing in strength across the world have had a negative impact on advocacy and coalition building in sub-Saharan Africa, restricting the space for debate and mobilisation, as well as funding for civil society (5, 6). Civil society organisations (CSOs) in the region are increasingly challenged to counter growing and dominant opposition with limited resources and capacity (7). Further, the effectiveness of CSOs can also be undermined by the short-term project-based nature of activities, linked to funding constraints and donor agendas, which can lead to reactive approaches with limited capacity and knowledge to make an impact for the long-term changes that are required (8–10). And the diversity of CSOs and competition for resources can lead to a dilution and fragmentation of civil society activities and advocacy in this space.

In this context, it is vital that CSOs supporting abortion law reform are able to frame debates in such a way as to mobilise public support and put the issue on the political agenda. In this article we analyse attempts to reform abortion laws in Madagascar, and the ways in which divisions in civil society both on issue framing and on mobilisation tactics may have impacted on the larger shared goal of law reform.

## Issue framing and the abortion debate

Issue framing is an essential component of advocacy and social mobilisation around public policy (11) both for placing policy issues on the political agenda, and for influencing the adoption of legislation. Framing is particularly important where policy issues are contentious and where issues of morality can be invoked (12) which is very strongly the case with regard to abortion. Research on framing of the abortion debate has shown how political opponents and social detractors have a major influence on ideologies of social movements and civil society, (13) as do the opportunity structures available (14). In the classic theories of framing and social movements, Snow and Benford (15) argue that consensus and social mobilisation are achieved by three frame alignment processes of diagnostic, prognostic, and motivational framing. In other words, CSOs or social movements must work to diagnose the problem in a way that resonates with potential supporters, propose a plausible and achievable solution through mobilisation, and issue a “call to arms” which motivates action. In their work specifically on the framing of abortion debates, McCaffrey and Keys (13), aim to expand the model proposed by Snow and Benford (15) and others (e.g., (16, 17), to include analysis of what they call competitive framing in cases of strong opposition between social movements and countermovements. They propose three strategies which are used by social movements in response to ideological challenges by opponents or countermovements which are polarisation-vilification, frame saving, and frame debunking. As we discuss further in the rest of the article, we found all these tactics used at different points by pro-choice reform activists.

Internationally, there are two clear and distinct framings for law reform amongst pro-choice advocates, although one is not exclusive of the other and reform movements may indeed mobilise both, often for different audiences or different circumstances. One is a women's rights argument and the other is presented as a public health concern. In the latter case, the argument is focused on removing criminalisation of abortion to save a woman's life. This framing of abortion reform in a medicalised way, as that of “saving women's lives”, is clearly present in global political debate were UN consensus documents have primarily focused on issues related to women's physical health to justify abortion and not addressed access to abortion as an issue of gender equality (18). In international policy discourses “a heavy emphasis is placed on the connection between restrictive laws and high rates of unsafe abortion, leading to maternal mortality and morbidity” (18), 29. This public health framing has filtered down from the international arena to many national debates, including that in Madagascar, as we discuss further below.

Another main thread of the pro-choice argument is framed in terms of women's rights and gender equality (19). This line of argument, which comes from a more explicitly feminist perspective rooted in the idea that “women's rights are human rights” (20), page 319, frames abortion as a right to bodily autonomy, “a woman's right to control her own body” (21), page 4. In the international arena, these positions have been severely challenged in recent years by the strengthening of “anti-gender” movements which are leading a backlash against the advances achieved in putting sexual and reproductive rights on the global health agenda (22).

Within this women's rights framing, pro-choice activists in Africa have used the international law arena to push for reform, and particularly the opportunity structure presented by the Protocol to the African Charter on Human and Peoples' Rights on the Rights of Women in Africa, (commonly referred to as the Maputo Protocol) which enshrines women's right to access comprehensive reproductive health care services and information on contraception, and safe abortion, without discrimination. Through the Protocol, State Parties are obliged to protect women's reproductive rights, particularly by authorising safe abortion in the cases listed in Article 14. 2 c. In addition, the Maputo Plan of Action (which operationalises the Continental Policy Framework on Sexual and Reproductive Health and Rights) urges Governments to adopt legal policies and frameworks to reduce cases of unsafe abortion, to develop and implement national action plans to mitigate the prevalence of unintended pregnancies and unsafe abortions.

It is also important to take into account discursive opportunity structures to understand the ways that social movement actors craft their frames in response to local political context and structures (23). As we discuss below, the Madagascan context is particularly restrictive in terms of political and discursive opportunities for talking about abortion and so proponents of reform must think carefully about how they can position themselves within this limited space.

## The Madagascan context

Madagascar has one of the most restrictive abortion laws in Sub-Saharan Africa with a total criminalisation of abortion, with no exception even to save a woman's life; Article 317 of the Madagascan penal code punishes “anyone by means of food, beverages, medicines, maneuvers, violence or any other means, procures or attempts to procure the abortion of a pregnant or supposedly pregnant woman, whether she has consented or not”^1^. Those found guilty may face large fines and imprisonment (24, 25). Anyone who assists a woman to procure or try to procure an abortion may face imprisonment of one to five years, and if they are found to have repeated the offence they can face up to ten years in prison. Women themselves who try to abort may be sentenced to up to two years imprisonment. As all abortion is illegal, post-abortion care is not openly provided, but may be accessed under another label. Women seeking post-abortion care prefer to try to do it through private hospitals as the quality of care in public hospitals may be of low quality and also carries an additional risk of criminal prosecution if abortion is suspected (26).

As is the case of other restrictive contexts, criminalisation has not led to a decrease in the practice of abortion in Madagascar, but merely that women resort to illegal and unsafe abortions (25). While maternal mortality due to complications arising from unsafe abortions is difficult to determine in Madagascar due to changing methodologies for measuring maternal mortality, in 2012 maternal mortality due to unsafe abortion was estimated to be 11.8% (27). Other estimates indicate that between 2015 and 2019, 42% of pregnancies in Madagascar were unintended and that 63% of these unintended pregnancies resulted in abortion^2^ (42). A study carried out in ten districts of Madagascar found that of those women who had an abortion, 27.7% sought post-abortion care for complications, indicating that these abortions were unsafe (28). Madagascar has one of the highest maternal mortality rates (392/100 000 in 2020) in the Southern African Development Community, with wide variation across the regions (29).

Previously there has been some space for Malagasy medical professionals to perform abortion in that the 1998 Code of Medical Ethics^3^ provided for therapeutic abortion if such an intervention was “the only means likely to safeguard the mother's life”, albeit on specific grounds such as the need for the opinion of two doctors and notification of the President of the Medical Council. However, this provision was discreetly removed from the Code of Medical Ethics in 2012^4^. At the time the removal was hardly noticed but as the debate over abortion has intensified, it has become more relevant, especially as health professionals have been targeted for prosecution for helping women to terminate pregnancy (30).

Abortion for therapeutic reasons (ie to save a woman's life) was once again put back on the table when a Reproductive Health and Family Planning bill was presented to Parliament in 2017, but efforts to include abortion in this bill failed when the Senate postponed the bill. Following the postponement, the article on abortion was removed on the grounds that it was to be discussed in a subsequent bill. The Reproductive Health and Family Planning Bill was subsequently passed but without abortion included^5^. Notably, the discussion around abortion over this time had negative impacts in that scrutiny had intensified over implementation of existing legislation. The Reproductive Health and Family Planning Bill now included text, in Article 28, that anyone practicing the termination of pregnancy for medical reasons will be punished with the penalties set out in Article 317 of the Penal Code. In 2018, the Ministry of Health sent a note to NGOs working in the area of sexual and reproductive health and rights stating that abortion in all its forms is prohibited in Madagascar and that they must take this into account in all of their activities in the country^6^.

In 2021, a Member of Parliament, with the support of civil society presented a new bill on amending and supplementing certain provisions of the Malagasy Penal Code and to make a case for abortions for medical reasons and for rape or incest^7^. However, the bill was not placed on the parliamentary agenda for debate. It was withdrawn by the Committee of Presidents ‘office on the grounds that this was not compatible with Madagascan values.

The different attempts to advance the issue of liberalisation of abortion, even to allow abortion for therapeutic reasons (ie to save a woman's life or in case of severe foetal abnormality), have been consistently blocked by political elites who have in turn come under pressure from religious leaders over this issue. The outcome has been an even tighter restriction of abortion, with documented cases of prosecutions of women and medical professionals who have sought or aided termination of pregnancy and for those found guilty, up to 3 years imprisonment.

Faced with this strong opposition to liberalisation of abortion laws, various national and international actors are still mobilised to try and effect reform, motivated both by concerns of gender equality and women's rights, and also by the real public health issue created by lack of access to safe abortion. However, these movements have become increasingly fragmented and arguments have arisen on how best to frame the issue to gain public and political support, and what mobilisation tactics to use. In this article we show how competing framings, not only between pro- and antichoice campaigns but also within the campaigns for abortion law reform, may undermine the ability of civil society to be effective in achieving *reform*. Having situated our argument in the context of the literature on issue framing, particularly in relation to abortion, we will go onto explain the competing framings which exist in Madagascar and how these have created division with the pro-choice movement which might be argued to have led to a failure to adopt abortion law reform. Arguably, the spotlight and debate around abortion have even created a more punitive environment for those who assist, provide or undergo an illegal abortion.

## Methods

The article is based on 20 key informant interviews conducted amongst a diverse range of stakeholders including representatives of civil society organisations, lawyers, journalists, religious and traditional leaders, health professionals, civil servants, and Members of Parliament in Madagascar from 2023 to 2025. Respondents were purposively selected in relation to their involvement in the debates over abortion law reform in Madagascar. Interviews were conducted in person by one or more of the authors, and were recorded when the interviewee consented to this. Interview duration was between 1 and 2 h. Interviewees were asked questions relating to their involvement in any campaigns or policy processes relating to abortion law reform in Madagascar, their opinions of such campaigns, and the barriers they perceived to achieving reform. All interview data was anonymised. Interviews were transcribed and a thematic analysis was carried out. We also reviewed relevant academic and grey literature which discuss sexual and reproductive rights in Madagascar, including academic articles, NGO reports, government reports and legislative texts which provided context and background to our interviews. Further, discussion of the central issues relevant to the paper took place in a workshop which brought together academics and various civil society organisations working on the issue of access to safe abortion in Africa which was organised in Durban, KwaZulu-Natal in South Africa in November 2023. For reasons of confidentiality we have anonymised our key informants and have not named the various civil society organisations who were interviewed or who are taking part in debates on abortion in Madagascar. Research ethics permission was obtained from the Comité Malgache d’Ethique pour les Sciences et la Technologie (CMEST).

Two of the authors of the article are researchers and activists based in Madagascar. The other two authors are researchers attached to a university in South Africa, and in France respectively. All authors contributed to the research, analysis and drafting of the article.

## Discussion and results

In what follows we discuss firstly how abortion law reform has been framed in public debate in Madagascar before going on to discuss the varying mobilisation tactics of the key actors in pushing for reform, and the ways in which differences in tactical and strategic approaches has led to a fragmentation in civil society mobilisations which can be argued to have made these less effective.

### Framing the abortion debate in Madagascar

In this first section we position the national debate on abortion in Madagascar within larger international debates, in order to situate the discursive and political opportunity structures which are available to those campaigning for abortion law reform in the country. The highly contentious political situation with regard to abortion law reform in Madagascar limits the number of pro-choice actors who are willing to talk about the issue. Interviewees for our study highlighted the fact that in this restrictive context, representatives of international organisations working in Madagascar have not taken a public position on the issue, with the exception of a regional UNFPA representative who made a statement when visiting Madagascar (the national UNFPA representative has not made any public statements on the issue): “We're in discussion with the government, about the laws that have been passed, to make sure that the protection of human rights is really respected. It's the same thing with abortion. As UNFPA, we don't say that abortion is a means of contraception. However, we do know that in certain circumstances, for women's rights, abortion should be offered. With our partners, we provide information on best practices in the region so that the government can make the best laws for its population”^8^.

The national space is limited by the power and influence of Churches in the country and the main opposition to abortion law reform in Madagascar comes from religious leaders who have a large influence over political elites. Nearly one third of the population are Anglican/Protestant FJKM/Lutheran (34.6%) and another third are Catholic (32.5%), with only one fifth (20.6%) of the population not belonging to a religious group (Institut National de la Statistique (31). The Council of Churches which is comprised of members of the four leading Church organisations in Madagascar (Catholic, Protestant, Lutheran and Anglican) has remained traditionally close to Madagascan Presidents and other political leaders, who are wary of pushing through any policies which will alienate these church leaders^9^. Sunday sermons focusing on anti-abortion rhetoric have also been noted in the country's growing number of Evangelical Churches which are mainly funded by conservative and right-wing American Evangelical Movements (32). One of our key informants pointed to the fact that since the overturning of Roe v Wade in the US, the finance for these Evangelical Churches and the “demonisation” of abortion has notably increased^10^. Churches have for a long time played a key role in Madagascan politics and culture, as Féron and Razakamaharavo (33), 366 explain: “Churches are present even in the most remote parts of Madagascar. They have radio and TV stations, they print newspapers, they have schools, they provide economic support to the deprived and, most importantly, Churches are generally considered as spiritual guides and even as “parents” to the population”. The influence of Church leaders on the abortion debate and the power they hold over political leaders was consistently highlighted in our interviews. Religion and religious discourses are invoked by opponents of abortion to defend “Malagasy values. “Parents” or “*ray aman-dreny”* is a term that incorporates both the physical and moral person who is understood to be a guardian of morals, customs, traditions and rules of good conduct in Malagasy society. In Madagascar, religious leaders, political leaders, traditional authorities, and other notables are also referred to as “*ray aman-dreny”,* as holders of authority in the society. However, this term has also been appropriated by some of those in power and employed as a device to assert their authority and impose their views. Further, access to the formal media by religious bodies was viewed as a powerful platform to leverage support of the communities. Pro-choice activists shared that they did not have free access to media and had to rely on social media which was limiting since many people in Madagascar do not have access to this^11^.

The notion of “culture” has been widely used as a discursive resource by opponents to abortion (34) and Madagascar is no exception. In Madagascar, as in various other African countries (35), the abortion debate is sometimes represented as invoking two opposed positions, one representing the decolonial defence of local culture and the other pushing a Western liberal women's rights framework. However, this understanding of a dichotomised debate is somewhat oversimplistic, and in reality, both sides of the debate can invoke elements of Malagasy culture to support their positions. Pro-abortion reform campaigners thus argued that abortion had long been part of life in Madagascar, pointing out that there was evidence that abortion had been practiced in Madagascar well before colonisation, with the first laws relating to abortion dating from the time of the colonialists in 1881 (36). To further demonstrate that abortion for therapeutic reasons is fully entrenched in Malagasy culture, it is common to say in the community that it is worth saving “the greatest life” (in this case the mother's) in the event of a complication during pregnancy or childbirth. Conversely, opponents draw on the view that children are highly valued and synonymous to a family's wealth. What precisely is meant by “Malagasy culture” is thus deployed in various ways depending on the hoped for political outcome.

Madagascar signed the Maputo Protocol in 2004 but campaigns for ratification since then have failed, blocked by political leaders because of Article 14 on abortion. However, whilst there is clear opposition to the ratification of the Maputo Protocol and whilst the Madagascan government has generally been reluctant to ratify the Maputo Protocol for reasons stated above, they are at the same time under pressure not to be one of the only countries in the region which has not ratified the Protocol, and thus signify their status as a women's rights outlier. Several key informants thus talked about the opportunity provided by the existence of the Protocol and the way that it could be used in advocacy. However, in Madagascar, as in other countries in the region, opponents of gender equality have depicted the Protocol as a “Trojan Horse” used by radical feminists, suggesting that introducing the more limited use of abortion for medical reasons is a pretext for then introducing abortion on demand (37), page 3. Some have even evoked a conspiracy inspired by Western feminists to push a radical agenda of abortion liberalisation in order to reduce the birth rate in Africa (37). Thus it is important to use arguments emanating from the international arena with some caution, and as one of our respondents explained, international NGOs need to “take a back seat” in public debates on abortion and allow local civil society to take the lead to reassure public opinion and fight back against this idea of international interference^12^.

Having outlined the main discursive and political opportunities available for framing pro- and anti-abortion campaigns at international and regional level, the remainder of the article will explore in more detail the actors campaigning for reform at national level in Madagascar and the ways in which they mobilise different strategies and framings to try and advance their cause.

### Who are the main actors pushing for reform?

Nationally, activists working for abortion law reform are divided into two main groups who, as we found, work in isolation from one another because of their competing framings and strategies as we discuss below. Originally, a larger coalition of CSOs grouped together under the umbrella of the Santé et Droits Sexuels et Reproductifs à Madagascar (Sexual and Reproductive Health and Rights in Madagascar) Network was formed at the initiative of five of the larger NGOs present in the country. However, it became quickly evident that this large coalition of 32 organisations^13^ did not agree on a number of fundamental issues, such as the need to campaign for liberalising abortion laws, or on the importance of comprehensive sexuality education in schools. As one of our interviewees explained, “we understood very quickly that we could not rely on this group for support and to get things done in support of abortion law reform”^14^.

Drawing on the McCaffrey and Keys (13) model of issue framing in abortion debates (described above) in the context of our research in Madagascar we found all of the three tactics they describe used at different points by pro-abortion reform activists, some pushing for a polarisation of the debate through outspoken and “radical” positioning, whilst others worked more on using scientific evidence to carefully debunk the framing of their opponents.

There are now two main groups pushing for reform, who have adopted notably different tactics and framing of the issue, with one side advocating for “a woman's right to choose” argument and calling for the immediate legalisation of abortion on demand, whilst the other group have adopted a longer-term strategy for the complete liberalisation of abortion, and are working in a more incremental way by framing their argument in terms of the need for therapeutic abortion to save women's lives. As we will discuss in the next section, these two groups have become increasingly polarised, which has resulted in a fragmented and sometimes unclear framing and strategy for abortion law reform in the country.

### Feminist or “non-feminist” mobilisation

The first group who were campaigning for abortion on demand firmly regarded themselves as “true feminists” based on the argument that abortion on demand is a basic women's right and positioned themselves as not being “feminist lite”^15^. The other group were, by extension, it was implied not feminist enough due to their more cautious, incremental approach in campaigning for abortion on specific grounds. As one of the leader's explained, they were campaigning for full legalisation of abortion on demand as “our principle is ‘my body, my choice”^16^. This stance had created some difficulties in collective organisation as for example it has resulted in one organisation no longer being able to talk to or work with the Madagascan Ordre de Médecins (Madagascan Doctors Professional Association). According to one of the group leaders this is because “they (the doctors) don't like the word feminist”^17^. The group acknowledged that they could perhaps be perceived as “too aggressive” but felt that it was important to stick to their principles and not to adapt their framing of the issue to a more “moderate” position, even if this created tensions with other groups in the pro-abortion law reform movement.

This is in contrast with another group of pro-choice advocates who also view themselves as feminists but who expressed the opinion that their framing and activism needed to be more adapted to the “socio-cultural” situation of Madagascar.^18^ They felt that if they argued for abortion reform on the grounds of gender equality and women's rights, they would be accused of importing “Western feminism” and going against “traditional” Malagasy values. As one member of this group explained, they decided to move towards framing the debate in terms of explanation of how abortion can save women's lives. They argued that they want to show the “human and positive” aspects of abortion law reform.^19^ This, they believed, could then lead eventually to a wider debate about women's choice, but this would be a longer term end goal, rather than an immediate way of framing the debate, especially since even abortion following rape or incest is still subject to debate: “At the moment, talking about women's choices is complicated and counter-productive. It's much better to talk about saving lives, about saving women's lives”^20^. This argument about framing the pro-choice debate in terms of women's health and saving lives was echoed by a journalist working at an African network on media, health and environment in Madagascar. He indicated that he felt it was important to talk about abortion but only in terms of women's health and saving lives. And he added: “being militant about women's rights is problematic and makes listeners angry”^21^. In contrast, what worked for gaining support according to this journalist was interviewing midwives who had experience of seeing women suffer serious complications or even die from unsafe abortions. Personal stories of women's suffering or death due to unsafe abortions were thus seen to be an alarm call to mobilise support for reform.

While one member of the latter group acknowledged there was a role for “radical” activism on the issue, they had explicitly developed a strategy of being “more moderate” in relation to the first more radical group who they believed were alienating public opinion with their women's rights stance.^22^ They also were viewed to have “hurt the cause” with doors being closed to some groups of activists as a consequence of the first group's actions^23^.

The difficulties in building unified feminist organisation and mobilisation have been demonstrated widely (38), and in the African context as Pereira (39), p.18 notes, the diverse context within which feminist activism takes place are ’still shaped by multiple layers of domination – imperial, colonial, military and/or authoritarian civilian rule’. And this means that ‘negotiating which strategies to adopt in achieving shared objectives is no straightforward matter’ ((39), p.18).

### Mobilisation tactics - behind the scenes or direct action?

There has also been disagreement amongst the pro-choice reform movements about the types of tactics to use, and particularly the appropriateness of street demonstrations. For example, in 2021 when the legislative proposal was brought to Parliament, one of the groups supporting it organised a street demonstration outside the National Assembly complete with large posters. Some of the organisers even suggested chaining themselves to the outside of the building to protest against the fact that the legislative proposal would not be debated. This was viewed as unacceptable behaviour by many commentators, even those who might have been favourable to the cause.

The way in which demonstrations were staged and slogans such as “my body, my choice” were displayed at meetings were said to further inflame the sensibilities of Members of Parliament and allies such as the Madagascan Doctors Professional Association. Part of public opinion identified the strategies adopted as being copied from the “extremist Western way of doing things”^24^ thus reinforcing the opposition rhetoric that abortion law reform is colonial and a Western import.

Other activists who didn't take part in the demonstration felt a more cautious approach was needed, and to instead adopt a “behind the scenes” method to try and find traction for their arguments at various levels of government before pushing for legal reform. One on one discussions with individual duty bearers and church leaders were seen as a productive way of lobbying and advancing the cause behind the scenes. One interviewee talked about how she had managed to obtain a meeting with a key government official where she was able to raise the issue of doctors being prosecuted for helping women to terminate pregnancy, and the impacts that this was having on the medical profession. She felt that this argument did have an impact even if the official could not openly call for legal reform in this area^25^. This quiet diplomacy was also the case for Church leaders, who other activists believed could be swayed by “ethics and science” and could be persuaded to keep their own counsel rather than voicing their opposition to therapeutic abortion. This kind of quiet diplomacy and behind the scenes advocacy could, activists argued, have a real impact in the longer term.

Members of this second group also called for more community level action not on mobilisation *per se*, but on education and information. They explained that even some women's civil society organisations were not in favour of abortion law reform because they did not fully understand the difference between therapeutic abortion to save a woman's life, and abortion on demand^26^. There was also reportedly concern amongst these same women's CSO's that discussing abortion would hurt their ability to raise other women's issues such as prevention of sexual and gender-based violence. Working to inform and educate on this issue, and use of more conciliatory tactics, could thus help to gain wider support amongst feminist movements for pro-choice advocacy.

### Fragmented movements lead to less effective action

A clear impact of the fragmentation of the pro-choice reform movement in Madagascar was revealed in which the presentation of the legislative proposal to reform the abortion law in 2021 unfolded^27^. A Member of Parliament who is very much identified with the pro-reform movement presented this legislative proposal, which attempted to amend the existing law to allow abortion in cases where pregnancy was a danger to the mother's life, or in cases of serious foetal abnormality and following a rape or an incest. In terms of CSO involvement, the process of supporting this legislative proposal was itself contested, as it was carried out without invitation (and the knowledge of) some pro-choice, feminist NGOs and civil society movements.

One stakeholder felt that the group supporting the development the legislative proposal on their own was a missed opportunity for greater involvement of like-minded civil society representatives to add weight to the discussion/debate: “We could have created momentum for reform if they had been more inclusive and brought in more different civil society organisations”^28^. As one of the respondents argued, presenting the legislative proposal in this way without first gaining wider support, including that of all the pro-choice movements, was a mistake as there was no “public base” supporting the reform^29^. In the face of well-organised opposition from the Council of Churches which made public statements on the proposed law before it was even debated in the National Assembly (a debate which in fact never took place), the fragmentation of the pro-abortion reform movement was thus a major disadvantage. As one interviewee remarked: “how can we be taken seriously by people if there is conflict within our own partnerships?”^30^ The legislative proposal has since been shelved, but the group promoting it explained that they were intending to bring it back to the National Assembly following legislative elections in 2024^31^.

### Moving forward in solidarity?

As Ferree (14), 339) argues, “framing is an interactive process that is inherently about inclusion and exclusion of ideas, so the choice of what ideas “the” movement endorses sets boundaries on its collective identity and on the definition of what losses would count as a movement failure.” In the Madagascan context this interactive process of frame alignment has led to a part of the pro-reform civil society moving towards a more pragmatic approach which does not directly oppose the anti-reform agenda, whilst another part of civil society has maintained its more radical and “feminist” stance. Both of the different strands of the movement are critical of their opponents’ approaches and what they include or leave out of their framing. This in turn has led to a split in pro-reform civil society which can be seen as damaging to the abortion reform cause, and perhaps to a failure of the movement. Although it can also be argued that the two competing framings have each contributed in some way to small advances in the movement.

While we present these groups as distinct, they are in reality both working towards a common goal and share many features. At the heart of it, both groups see themselves as feminist and share a common cause in seeing abortion law reform implemented in Madagascar. Indeed, individual positions in each group are themselves not fixed, with group members constantly reflecting on their positions and what tactic works best. In the second group, for example, one member shared that while she chose a less confrontational approach, she also felt that *any means* adopted by pro-choice advocates were needed and useful in working towards decriminalisation of abortion. The same informant also questioned the impact of their own group's approach (ie to request therapeutic abortions to save a woman's life) which failed to gain support from the public and “received the same contemptuous rejection as abortion (on demand)”. This participant felt that perhaps then they should stop being cautious and be more direct.

The negative perceptions which can be associated with the label “feminist” or “feminism”, negative perceptions which have only been reinforced with the growth of anti-gender movements, have had important consequences for how different strategic positions are viewed in Malagasy society. One key informant told us, for example, that the concept of feminism is generally negatively perceived and poorly understood in Madagascar. In light of the recent colonial past, feminism is seen “as a fundamentally Western and therefore foreign demonstration”^32^. The reluctance for some CSOs to openly claim to be feminist, therefore, stems from concern around how feminism might be understood in the local context as being a “Western import” and associated with the desire for women to dominate men. This means that even those who may consider themselves privately as feminists, might be reluctant to claim this label overtly within their campaigning. To return to the idea of Malagasy culture and education, Andriamanondehibe (40) highlights the importance of the “values of “henatra” (modesty), “hasina” (sacredness), “fahendrena” (wisdom, circumspection), without forgetting the predominant religious principles” and calls for a “Malagasy feminism” which is “respectful of the particularity of the Malagasy identity”.

## Limitations

We have attempted to mobilise a conceptual framework on issue framing and agenda setting in public policy to analyse and understand the ways that civil society has played a role in moblising for abortion law reform in Madagscar, and also the possible weaknesses in this mobilisation due to fragmentation and strategic differences. Limitations to our work include the fact that most of the respondents were part of the abortion access reform mobilisation and we had fewer interviewees who were against such reform. This could be an important avenue for further investigation.

## Conclusion

Divisions in pro-abortion campaigns are not limited to Madagascar but are part of a wider global problem of how to frame this issue in a context of increasing opposition. As Klugman (41), 14 argues, “just as the debate on the acceptability of sex work and whether sex work can be distinguished from trafficking has split the feminist movement and provided allies for those who are fundamentally against women's equality and rights; so we may find increasing dissent on the nuances of the question of abortion access, further weakening clarity of solutions and messages put forward by the movement for abortion access”. This is evident in the Madagascan context in the division between those advocating for complete legalisation of abortion on demand and a more limited legalisation of therapeutic abortion.

Our research in Madagascar shows two distinct strategic framings by different parts of the pro-abortion reform movement, one employing a consciously “radical” stand as feminists standing for women's right to choose, whilst the other group chooses to find a framing that they believe resonates more with existing public opinion, that of saving women's lives. Both groups criticise the other - one for not being “radical” or “feminist” enough, the other for being too “disruptive” and ignoring local sensitivities. The two sides of the movement have also disagreed on tactics, with one group pursuing more disruptive tactics for gaining visibility, whilst the other group prefers a more gradual approach attempting to find common ground and build the largest coalition possible. This fragmentation can be seen as damaging to a relatively small movement in a very restricted context. In this context, one group's action can directly impact the work of the other group, arguably with the risk of harming the cause of abortion law reform by putting the spotlight on the issue, and crystallising opposition, leading to increasing threats against women and health professionals and others who assist abortion. It can thus be argued that the timing of the presentation of the above-mentioned law amendments can be seen as a failure to assess the existence or not of a real opportunity structure, and to ensure issue framing that resonated widely.

However, there is also an argument that there is room for different strategies and framings even within so restrictive an opportunity structure. Whilst one group might be seen as too “radical” or “disruptive” they have also managed to gain large scale attention and debate for the issue of abortion. It could also be argued that this created some space for the other group to improve their standing by positioning themselves as more moderate and sensitive to local concerns and thus to be able to advance more discreetly in its behind the scenes lobbying. As Ferree (14), p339 asserts “Just who is a radical depends on the discursive context in which they speak.” In restrictive contexts, it is indeed difficult to know when is the best time to act, and opportunity structures are clearly limited. Opening up debates and forcing the agenda in these circumstances can result in the opening of a Pandora's box where a limited hope for change co-exists with an array of possible negative consequences. These issues are clearly not unique to Madagascar and solutions can be found through regional exchange and learning in effort to build effective coalitions around a shared goal. To do this, it will be important to explore regional opportunity structures that provide space for constructive dialogue.

## Plain language summary

This article examines the debates over abortion law reform in Madagascar. We analyse the way that the arguments have been framed by different actors entering into the debate, and the difficulties involved in uniting different actors all pushing for reform. We found that whilst various civil society movements are organising to lobby for reform, their tactics differ significantly which may cause conflicts between them and impact on the effectiveness of the movement for abortion liberalisation.

## Data Availability

The raw data supporting the conclusions of this article will be made available by the authors, without undue reservation.

## Footnotes

^1^Madagascar Code Pénal du 17 juin 1972 mis à jour au 30 juin 1998

^2^ https://www.guttmacher.org/regions/africa/madagascar

^3^Décret n° 98-945 du 04 novembre 1988 portant Code de déontologie médicale

^4^Decret N° 2012- 0632 du 13 juin 2012, portant Code de déontologie médicale

^5^Loi n° 2017-043 sur la Santé de la Reproduction et la Planification Familiale

^6^Interview, 30 October 2023

^7^Madagascar: Law on therapeutic termination of pregnancy again rejected by the Assembly (rfi.fr)

^8^Statement of the Regional UNFPA Representative during her visit in March 2024. https://midi-madagasikara.mg/unfpa-lydia-zigomo-dans-nos-murs/

^9^Interviews held on 30 October 2023, 27 June 2024.

^10^Interview 30 October 2023.

^11^Interview 1 November 2023.

^12^Interview 31 October 2023

^13^Including international NGOs, local NGOs, and various women's rights and human rights associations

^14^Interview 28 June 2024

^15^Interview 2 November 2023

^16^Interview, 2 November 2023

^17^Interview, 2 November 2023

^18^Interview, 28 June 2024

^19^Interview 30 October 2023

^20^Interview 30 October 2023

^21^Interview 31 October 2023

^22^Interview 31 October 2023

^23^Interview 2 November 2023

^24^Comments from social media users about the street demonstrations

^25^Interview 31 October 2023

^26^Interview 31 October 2023

^27^ *PROPOSITION DE LOI n°004- 2021/PL modifiant et complétant certaines dispositions du Code pénal malagasy*

^28^Interview 31 October 2023

^29^Interview 30 October 2023

^30^Interview 30 October 2023

^31^Interview 3 November 2023

^32^ https://lexpress.mg/23/01/2017/il-suffit-dune-crise/
